# Comment on article “A Simple Classification of Pancreatic Duct Size and Texture Predicts Postoperative Pancreatic Fistula: A Classification of the International Study Group of Pancreatic Surgery”

**DOI:** 10.1097/AS9.0000000000000378

**Published:** 2024-01-23

**Authors:** Jinlong Hu, Yanfei Yang

**Affiliations:** From the *Department of General Surgery, The First Affiliated Hospital of Dalian Medical University, Dalian, China.

We have recently been very interested and discussed in depth an article entitled “A Simple Classification of Pancreatic Duct Size and Texture Predicts Postoperative Pancreatic Fistula: A Classification of the International Study Group of Pancreatic Surgery.”^1^ The authors retrospectively performed a systematic literature search and identified risk factors for postoperative pancreatic fistula using a meta-analysis of clinical related-postoperative pancreatic fistula (CR-POPF) rates for pancreatic texture (soft vs. nonsoft) and main pancreatic duct (MPD) diameter using the Mantel–Haenszel method.^1^ According to the results of the study, an updated classification based on MPD and pancreatic texture was proposed, and it was determined that a soft pancreatic landmass and an MPD diameter ≥3 mm significantly increased the risk of CR-POPF.^1^ We are very grateful for the research done by the author, but there are still some deficiencies that we need to discuss together.

First of all, the authors analyzed the risk factors and finally screened out MPD and pancreatic texture as the basis of classification, and I think that these screened-out risk factors need to be discussed.^1^ According to relevant studies, pancreatic thickness (PT) is also a high-risk factor for CR-POPF.^2^ Moreover, the authors of this article did not show the statistical significance of pancreaticoduodenectomy in the article, so the inclusion of pancreaticoduodenectomy in the classification basis can be a better classification and assessment of postoperative pancreatic fistula.^2^ Meanwhile, there are also related studies reporting that MPD/PT is also a risk factor for CR-POPF^3^ and better evaluating the scientific rigor, standardization, and credibility of this classification, as well as the scientific basis of this classification, through the perspective of pancreatic ratio, and at the three-dimensional level.^2,4^ The scientific, rigorous, standardized, and credible basis of classification has been improved accordingly.

Last but not least, the main theme of the authors of this article is simple categorization, and I believe that based on the reported results of relevant predictive models of CR-POPF in recent years, we can learn that the predictive results, screening of risk factors, clinical portability, and clinical reproducibility are relatively low, and the model fit is not too good.^3,5^ Therefore, it is important to classify, screen, include, and exclude risk factors in a more scientific, rational, and logical way, so as to facilitate further research on the CR-POPF model and better guide clinical practice.^5^ Finally, we would like to thank the authors again for their contribution. This study provides a further reference to CR-POPF prediction studies from a new classification basis, and its significance deserves further investigation.
